# Differential cohesin loading marks paired and unpaired regions of platypus sex chromosomes at prophase I

**DOI:** 10.1038/s41598-017-04560-5

**Published:** 2017-06-26

**Authors:** Aaron E. Casey, Tasman J. Daish, Jose Luis Barbero, Frank Grützner

**Affiliations:** 1 The Robinson Research Institute, School of Biological Sciences, the University of Adelaide, South Australia Adelaide, Australia; 2 Centro de Investigaciones Biologicas (CSIC)/Ramiro de Maeztu, 9 28040 Madrid, Spain

## Abstract

Cohesins are vital for chromosome organisation during meiosis and mitosis. In addition to the important function in sister chromatid cohesion, these complexes play key roles in meiotic recombination, DSB repair, homologous chromosome pairing and segregation. Egg-laying mammals (monotremes) feature an unusually complex sex chromosome system, which raises fundamental questions about organisation and segregation during meiosis. We discovered a dynamic and differential accumulation of cohesins on sex chromosomes during platypus prophase I and specific reorganisation of the sex chromosome complex around a large nucleolar body. Detailed analysis revealed a differential loading of SMC3 on the chromatin and chromosomal axis of XY shared regions compared with the chromatin and chromosomal axes of asynapsed X and Y regions during prophase I. At late prophase I, SMC3 accumulation is lost from both the chromatin and chromosome axes of the asynaptic regions of the chain and resolves into subnuclear compartments. This is the first report detailing unpaired DNA specific SMC3 accumulation during meiosis in any species and allows speculation on roles for cohesin in monotreme sex chromosome organisation and segregation.

## Introduction

Cohesins are non-histone proteins of the structural maintenance of chromosomes (SMC) group. Traditionally they are described as the molecular glue that keeps sister chromatids physically attached after DNA replication during both mitosis and meiosis. More recently it has been discovered that cohesins are also central to a number of other fundamental mechanisms in chromosome biology (Reviewed by Mehta *et al*.^1^) including transcriptional regulation^2, 3^, DNA double strand break (DSB) repair^4, 5^, chromosome condensation^6, 7^, DNA replication^8^, homologue pairing and recombination^9–11^ and synaptonemal complex (SC) formation^12–15^. Cohesins have also been implicated in promoting nucleolar structure and function^16^.

Mitotic cohesin is a multiprotein complex comprising four subunits: SMC3, SMC1α, an α-kleisin subunit (Rad21), and a sister chromatid cohesin component (Stromal Antigen 1 or Stromal Antigen 2). There are also meiosis specific isoforms including SMC1β, Rad21L and Rec8 (α-kleisin subunit isoforms), and the sister chromatid cohesin subunit isoform Stromal Antigen 3 (SA3/STAG3). Although many of the meiosis specific functions remain unclear it is known that some meiotic cohesin subunits appear to act in functionally specific combinations^10^. While most cohesins are involved in sister chromatid cohesion, some, such as the RAD21L containing complexes, function in non-sister chromatid cohesion between homologous chromosomes^10^. One significant common feature between the different cohesin complexes (CCs) is the inclusion of the SMC3 subunit.

In meiotic cells, immunostaining for cohesin complexes shows the same localisation as the SC to the extent that some investigations now report the CC to be part of the SC^15^. In mouse, RAD21L is recruited to the chromatin loops of the XY sex body, a pattern similar to the ɣH2AX repressive histone variant which marks silenced sex chromatin^11, 17^. Another aspect of meiotic sex chromosome regulation involves an association with nucleoli which has been implicated in their organisation and transcriptional silencing due to the close association at pachytene^18^. Proteins that localise to the sex chromosomes during pachytene have also been observed to accumulate in nucleoli which may provide some insights into meiotic nucleolar function^19, 20^. During mouse zygotene, nucleoli form on nucleolar organising regions (NORs) however at pachytene these nucleoli disassociate from the NORs, coalesce and migrate to the sex body until diplotene^21^. While similar nucleolar-sex chromosome organisation has been reported in multiple eutherian species^22–24^, metatherians^25^ and even invertebrates^26^, the purpose of this association remains unclear.

The platypus carries a highly unusual sex chromosome system comprising 5X and 5Y chromosomes^27, 28^ with seven known pseudoautosomal regions (PARs; X_1_Y_1_, Y_1_X_2_, X_2_Y_2_, Y_2_X_3_, X_3_Y_3_, Y_3_X_4_ and Y_4_X_5_) that mediate pairing to form an alternating XY 10 sex chromosome chain during meiosis I^27, 29, 30^. The complexity of monotreme sex chromosomes and their homology to the avian Z raises fundamental questions about the mechanisms underlying meiotic pairing, recombination, evolution of meiotic sex chromosome inactivation (MSCI) and sex chromosome segregation. Our recently published findings indicate that the platypus SC may be different to other mammalian SCs with 3 significantly divergent copies of *SYCP3* which are expressed at high levels in platypus testis^31^. However, there is currently no information about the role of cohesin proteins in platypus meiosis.

In the present study we examine cohesin and synaptonemal complex dynamics during meiotic prophase I in platypus, with particular focus on the sex chromosome chain. We discovered a highly dynamic and specific reorganisation of accumulated cohesin associated with the formation of the sex chromosome complex and to a large nucleolar structure. Our finding of temporal and differential cohesin accumulation specifically on asynapsed sex chromosome chromatin loops implicates a possible role for cohesin in sex chromosome organisation at prophase I. Specifically we speculate roles in unpaired DNA-specific functions and/or establishment of configurations or markers for faithful alternate segregation of the 10 sex chromosome elements at anaphase I.

## Results

### At meiotic prophase I platypus sex chromosomes accumulate SMC3 while in close proximity to a large nucleolar body

In order to investigate the dynamics of SC formation in monotreme prophase I, we used antibodies raised against the central element protein SYCP1 and the cohesin subunit SMC3 to visualise chromosome axial cores. SMC3 is a component of all meiotic cohesin complexes discovered to date and platypus SMC3 is highly conserved with over 99% pairwise identity with human SMC3 at the amino acid level (Figure S1). Western blotting using platypus testis whole cell lysates identified a single protein species of the expected size (Figure S2).

**Figure 1:**
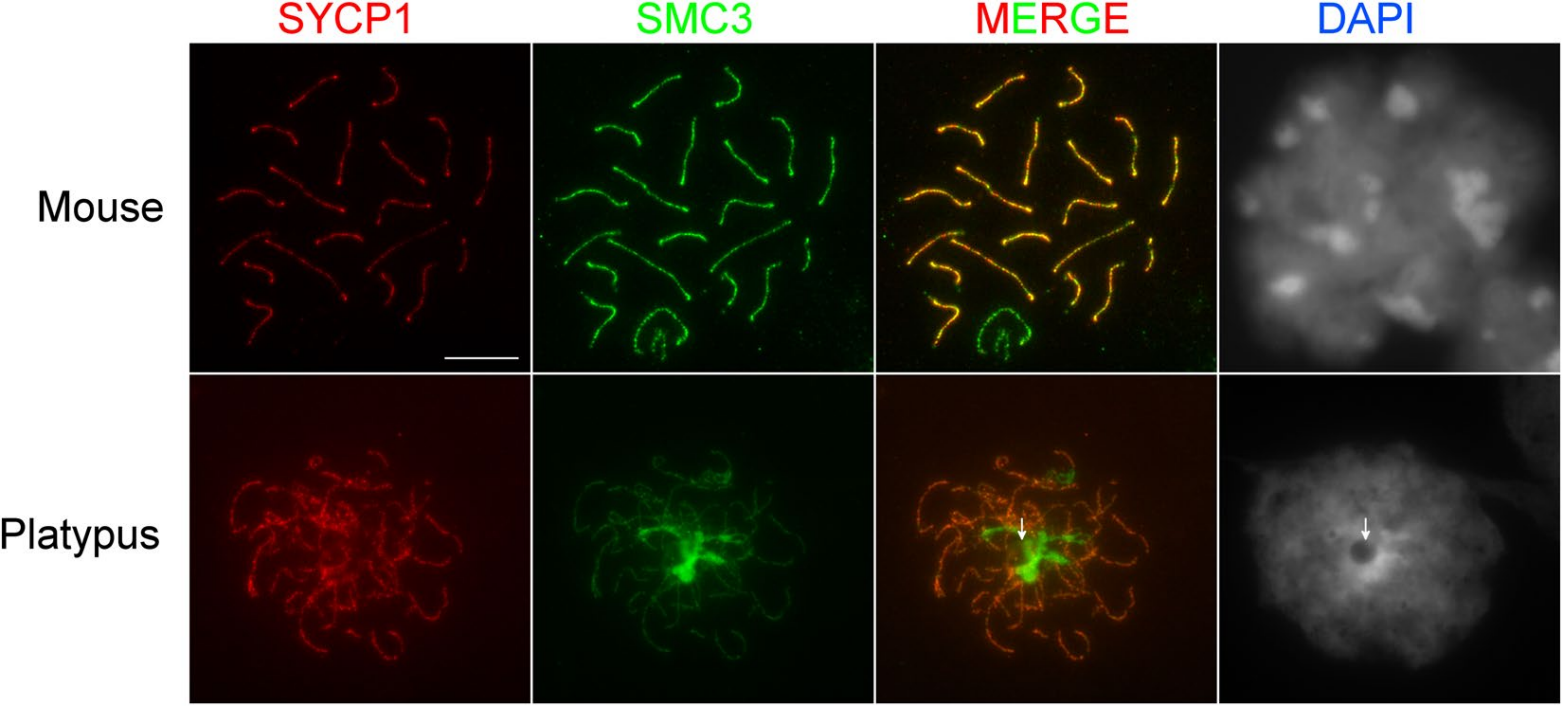
SMC3 and SYCP1 localisation in mouse (upper panel) and platypus (lower panel) pachytene cells. SMC3 localises to the axial core of all chromosomes while SYCP1 localises only to synapsed chromosomes, thus, the sex chromosomes appear as the only chromosomes exhibiting axial core signals without a central element signal. While the mouse signal on the sex chromosomes is as expected from published work, the signal in the platypus showed a significantly higher level of SMC3 recruitment on regions without a corresponding axial signal. This region also consistently associated with a large, spherical, DAPI poor region of the cell (white arrow). Scale bar = 10 µm.

**Figure 2:**
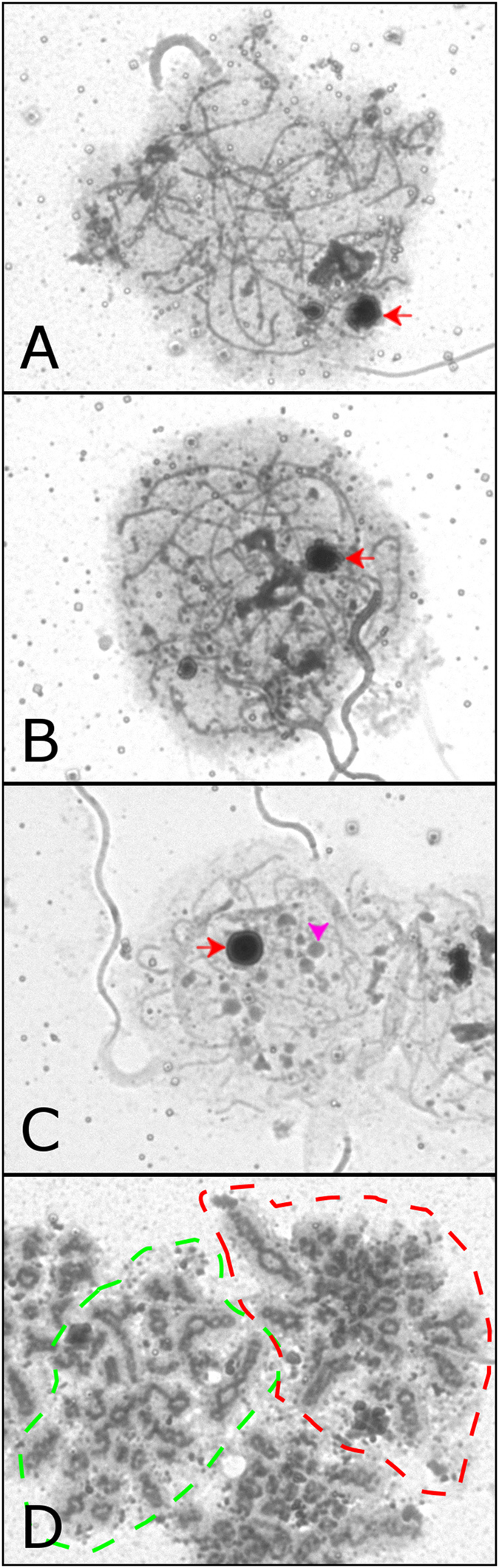
Silver staining of platypus pachytene cells. From zygotene (**A**) to diplotene (**C**) there is a heavily stained large spherical structure that is the nucleolus (red arrow). In diplotene many smaller spherical bodies are visible, usually four larger bodies (examples marked with large pink arrowheads) and numerous smaller bodies (examples marked with small pink arrowheads). By diakinesis (**D**) (two cells are outlined by red and green dashes), there is no obvious nucleolus and the axial core clearly has significant protein recruitment, having a darker core than seen in any of the other stages. Scale bar = 10 µm.

SYCP1 immunostaining showed an expected SC staining pattern in platypus prophase I cells, however the SMC3 antibody detected remarkably strong localised accumulations unlike mouse cells at the same stage (Fig. 1). To confirm this pattern as representing cohesin complex distributions we immunostained for the meiosis specific cohesin STAG3 using a custom antibody which showed identical staining patterns described for SMC3 (Figure S3). As this antibody had limited availability and mimicked the accumulation patterns of SMC3 we used a commercial anti-SMC3 antibody for the remaining cohesin complex assessments. We also noted that the focus of strong SMC3 accumulation was consistently in close proximity to a large DAPI negative structure (Fig. 1, arrow). Silver staining yielded intensely stained regions of the same size and shape as the large DAPI void (Fig. 2, red arrow) showing the proteinaceous nature of this structure. Our recent findings that this large DAPI void physically associates with the NOR bearing platypus chromosome 6 and with the sex chromosome chain at pachytene^32^ suggests that this is a large nucleolus.

**Figure 3:**
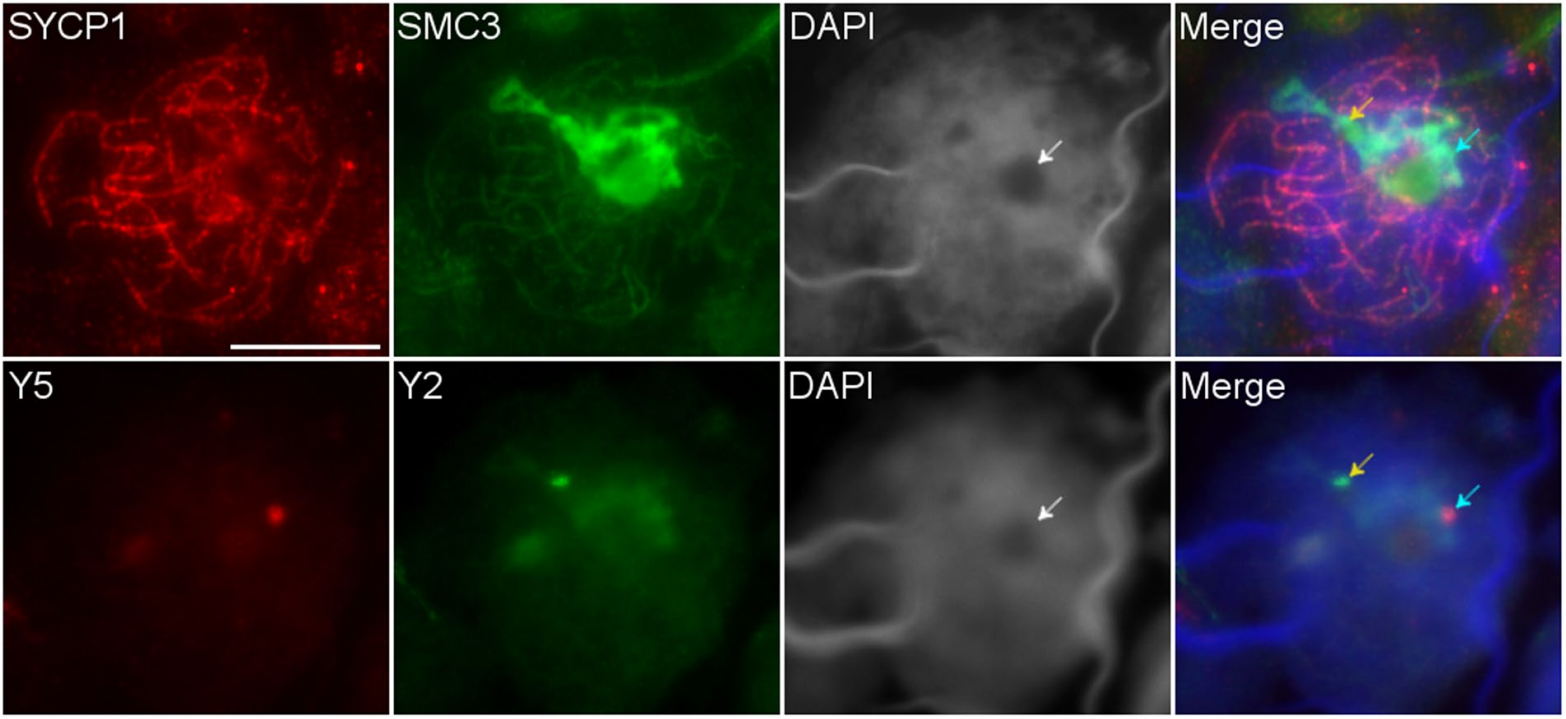
Colocalisation of sex chromosomes with SMC3 accumulation in platypus pachytene cell nuclei. Y specific probes; Y_2_ (yellow arrow) and Y_5_ (green arrow). The region of SMC3 accumulation is also associated with a large DAPI poor region (white arrow). Scale bar = 10 µm.

Pachytene sex chromosomes can be identified as the only chromosomes with an axial element signal (SMC3) which also lacks a central element signal (SYCP1). This is because the bulk of the sex chromosome DNA is not homologous and therefore doesn’t undergo SC-mediated synapsis involving SYCP1. As the intense SMC3 accumulations in platypus prophase I nuclei lacked SYCP1 this represented the location of sex chromosomes. This was confirmed by immuno-FISH on meiotic spreads using anti-SMC3 and anti-SYCP1 antibodies followed by two Y chromosome specific BAC probes targeting Y_5_ (Oa_Bb-152P15) and Y_2_ (CH236-145P9). Y_5_ and Y_2_ consistently reside within the regions of SMC3 accumulation, demonstrating that the platypus sex chromosomes experience significantly more cohesin loading in prophase I relative to the autosomes (Fig. 3). The autosomal SMC3 loading appeared similar between platypus and mouse however this proved difficult to demonstrate using identical exposure times as the massive SMC3 signal in platypus saturated the image and obscured the surrounding autosomal element resolution.

### Cohesin accumulation on sex chromosomes is highly dynamic through prophase I

**Figure 4:**
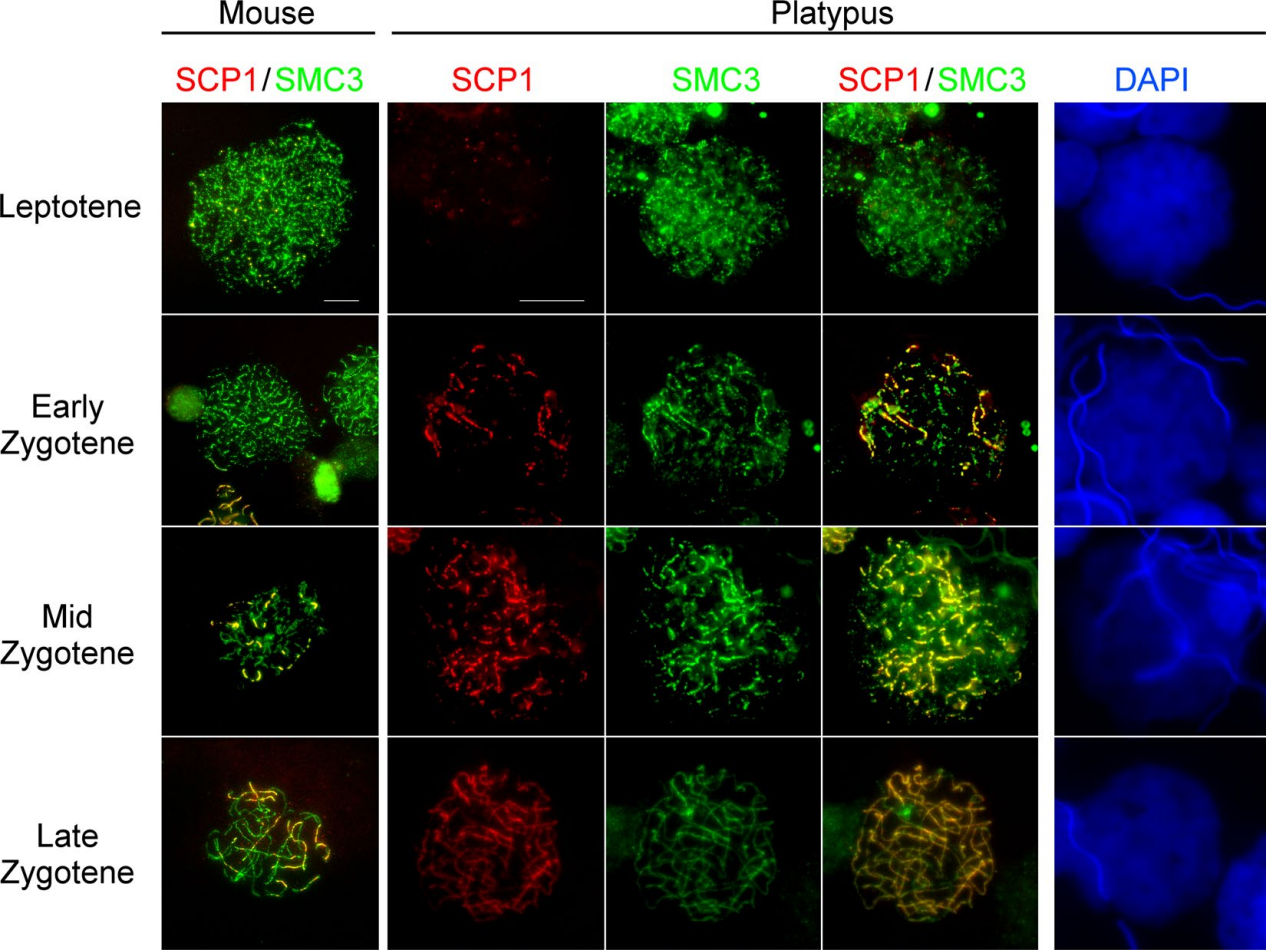
Immunolocalisation of SMC3 and SYCP1 in mouse and platypus early prophase I cells. In leptotene, both platypus and mouse cells exhibit a homogenous speckling of cohesin throughout the nucleus. By mid zygotene in mouse, many axial elements are visible with only a few with a partial central element. In contrast to this, in platypus most axial elements also have a central element. In late zygotene the giant nucleolus begins to form (white arrow). Note that mouse and platypus stages are provided for comparison and may not represent exactly equivalent stages. Scale bar = 10 µm.

**Figure 5:**
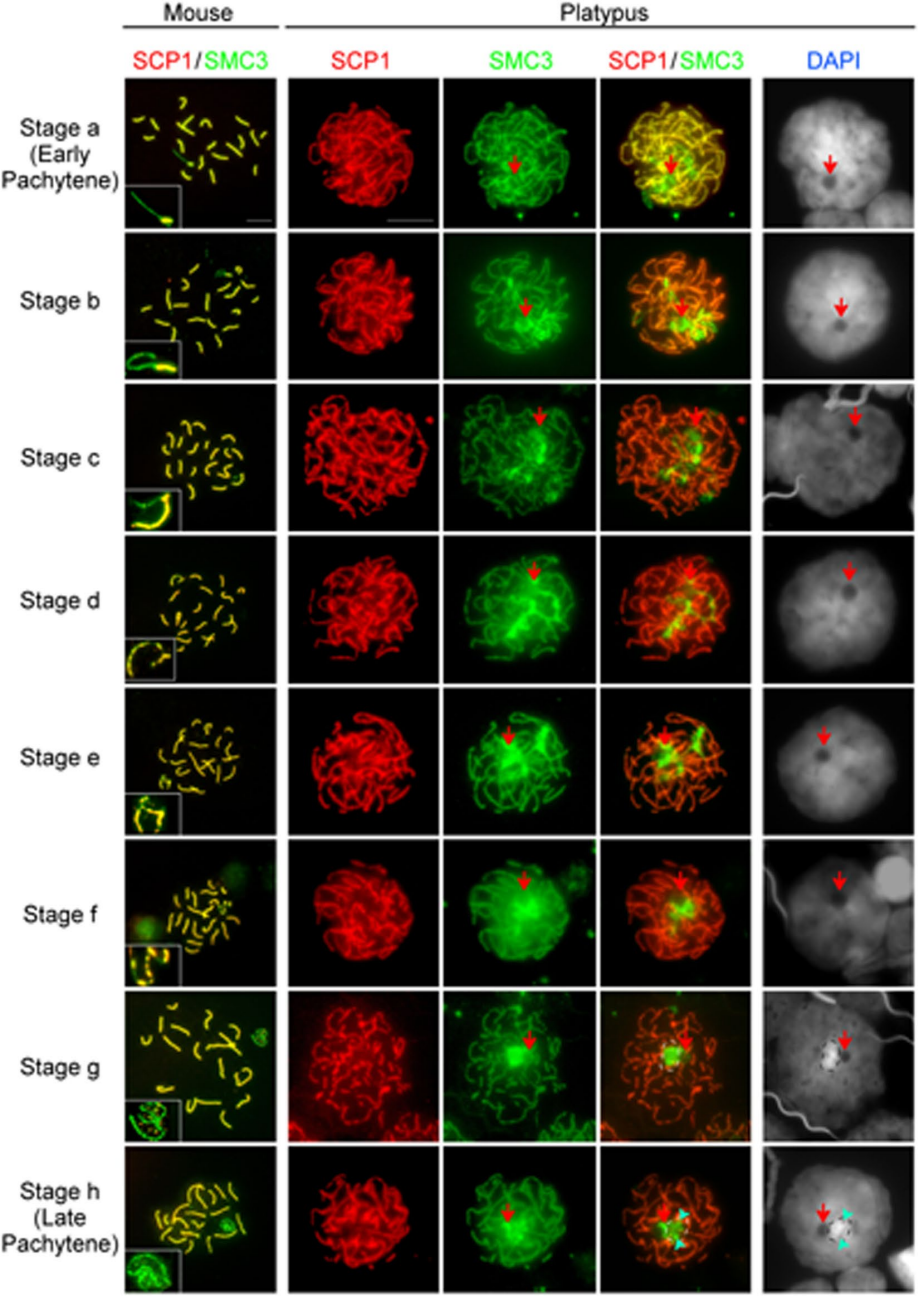
Substages of mouse and pachytene cells based on their sex chromosome configuration and synaptonemal complex length. There are eight distinct mouse sex chromosome patterns for each stage (insets). In all stages the platypus sex chromosome chain is attached to a large nucleolus (red arrow). In platypus stage g and h nuclei, the region of significant cohesin accumulation colocalises with a DAPI intense region (black/white dashed surround). In platypus late pachytene, protein bodies are observed (light blue arrowheads). Note that mouse and platypus stages are provided for comparison and may not represent exactly equivalent stages. Scale bar = 10 µm.

**Figure 6:**
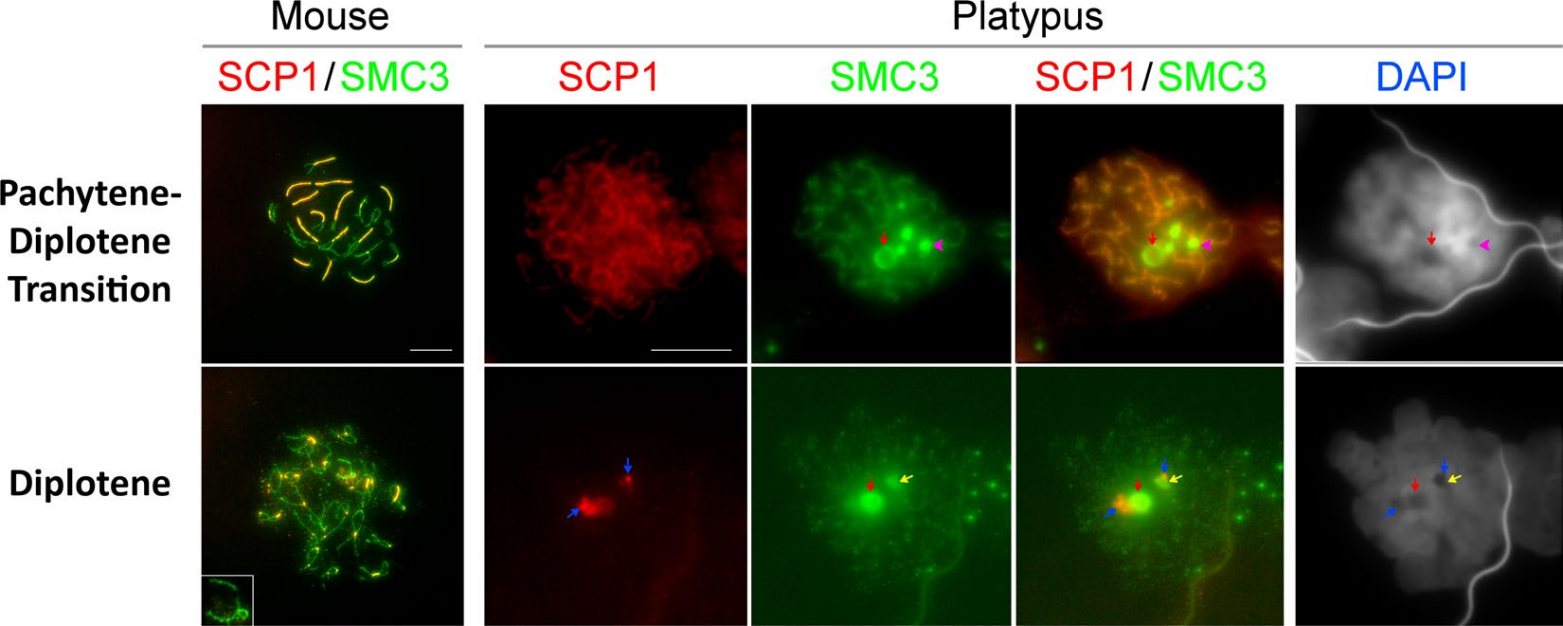
Pachytene-diplotene transition and diplotene in mouse and platypus prophase I cells. In platypus diplotene, four large protein bodies (large pink arrow heads) are observed and the large nucleolus persists (red arrow). In mouse diplotene cells, only the sex chromosomes have SMC3 staining at the axial core, while the staining on autosomes is significantly reduced to foci spread along the axial cores. In platypus there is only weak SMC3 staining scattered on the chromosomes. Also in platypus, there is a larger region that contains SMC3 (red arrow) and a smaller region that contains SMC3 (yellow arrow). Both regions also have a smaller associated region containing SYCP1 (blue arrows). Note that mouse and platypus stages are provided for comparison and may not represent exactly equivalent stages. Scale bar = 10 µm.

In our original experiments, we observed a spectrum of SMC3 staining patterns in platypus prophase I cells and binned these configurations to identify leptotene, three substages of zygotene, multiple pachytene substages, and diplotene. For comparison, we identified similar substages in mouse based on previous work^33, 34^ (Figs 4–6).

#### Identified prophase I substages based on SMC3 configurations

*Leptotene*; Both mouse and platypus early prophase I stage nuclei lack SYCP1 staining as it precedes commencement of synapsis. During this stage we observed a homogeneous speckling of SMC3 signal distributed throughout the nucleus (Fig. 4, leptotene panel).

*Zygotene*; In early zygotene in both mouse and platypus, the axial elements begin to form, however in platypus, there was an associated SYCP1 signal in most cases, while there was no such signal at this early stage in mouse. This was more pronounced as zygotene advanced, with most axial elements in platypus having a corresponding central element, in contrast, mouse axial element formation preceded central element assembly as previously reported^35^ (Fig. 4, zygotene panels).

*Pachytene* Stage a; in early pachytene the sex chromosome chain is evident as a long thin element which at first (Fig. 5, stage a) is only discernible via double immunostaining with SYCP1 and SMC3 and appearing as the only chromosomal elements not containing central elements. At this stage one end of the chain appears to be associated with the nucleolus.

*Pachytene* Stage b; the sex chromosome chain maintains a conformation consistent with stage a, however it has a marked increase in the amount of SMC3 localising to the chain such that it can be identified with SMC3 immunostaining alone (Fig. 5, stage b).

*Pachytene* Stage c; folding of the sex chromosome chain is apparent at multiple points (Fig. 5, stage c) signalling commencement of the stage of chain contraction toward the nucleolus.

*Pachytene* Stage d; a central region of the chain appears to now associate with the nucleolar body (Fig. 5, stages d).

*Pachytene* Stage e; at stages a and d, two regions of the chain associate with the nucleolus. At stage e, the portion of the chain between these two regions contracts and also becomes associated with the nucleolus.

*Pachytene* Stage f; the last regions of the chain are pulled toward the nucleolus. The sex chromosomes are not yet heterochromatic and some twisting of the chain elements can still be observed.

*Pachytene* Stage g; the entire condensed chain is located adjacent to one hemisphere of the large nucleolar body and the cohesin loading now appears to homogenously cover the area occupied by the contracted sex chromosome chain. The sex chromosomes colocalise with an intense DAPI signal indicating condensation of the chromatin (e.g. Fig. 5, stage g).

*Pachytene* Stage h; at late pachytene we observed formation of small round DAPI negative SMC3 positive spheres (Fig. 5, stage h) of similar configuration to the protein accumulations observed in the silver staining (Fig. 2, panel C pink arrows/arrowheads). This suggests that SMC3 is rapidly removed from the sex chromosome chromatin at late pachytene and subsequently is concentrated into DAPI negative proteinaceous foci. The sex chromosomes reside in this region that is generally still heterochromatic.

**Figure 7:**
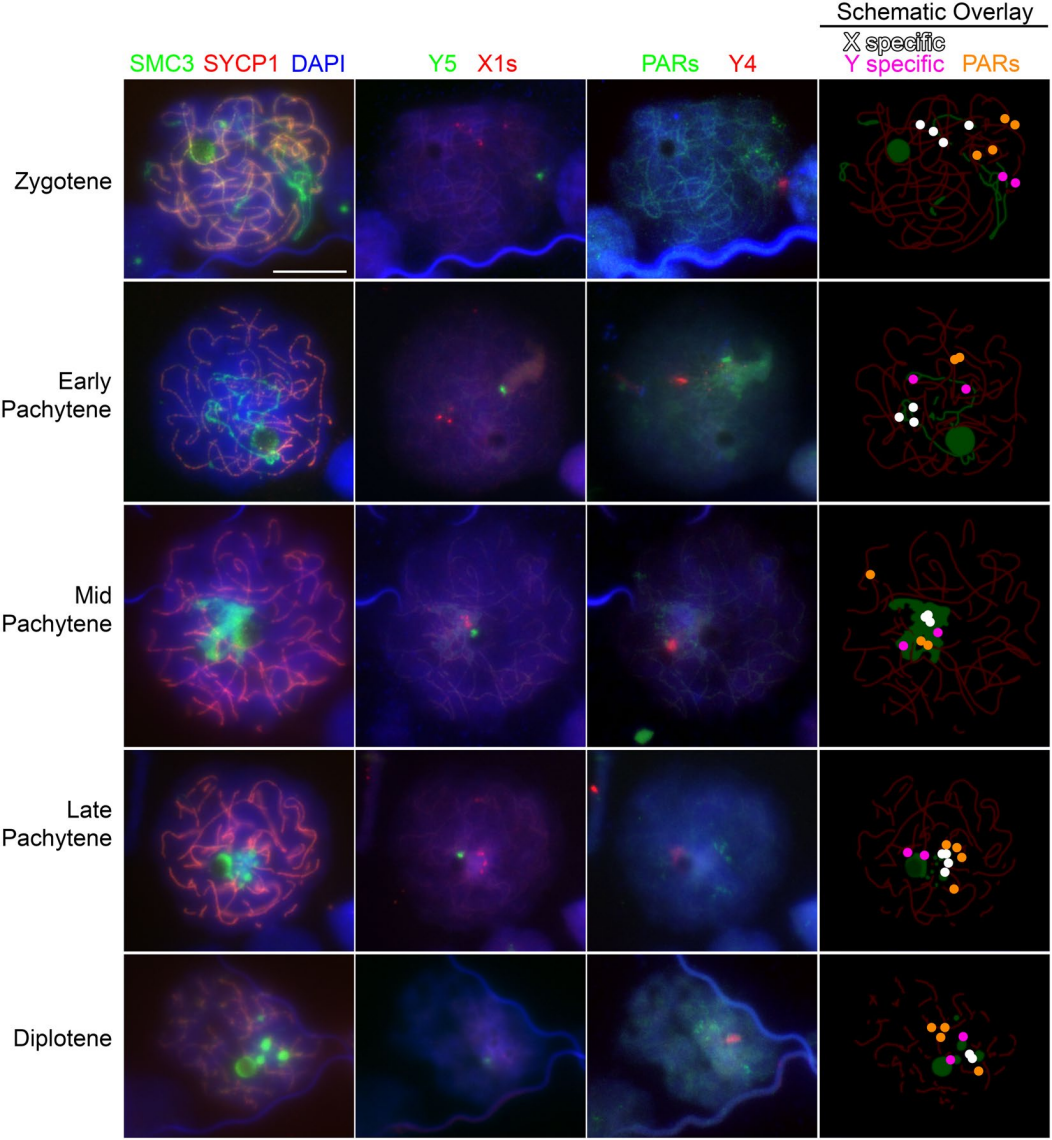
Sex chromosome localisation during prophase I. Four X_1_ probes, a Y_5_ probe, a Y_4_ paint and four PAR probes are visible in each of the stages. From zygotene to pachytene, the X and Y specific probes localise to regions that have only SMC3 labelling, but no central element staining. Importantly in mid pachytene, no X or Y specific signal extends to a region outside of that which has increased SMC3 accumulation. In contrast, the PAR probes consistently localise outside of this region and localise over, or adjacent to, central elements. In diplotene, the X and Y specific probes localise to regions adjacent to, but not within, protein bodies containing SMC3, while the PAR probes localise to regions over, or adjacent to, central elements. Scale bar = 10 µm.

*Pachytene to diplotene transition*; The sex chromosomes begin to spread out and initially, small SMC3 positive bodies increase in number with up to five large (Figs 2 and 7, pink arrowheads) and numerous small foci visible during diplotene (Figs 2 and 7, pink arrows).

*Diplotene*; The SMC3 has resolved to a few larger foci (Fig. 6). The chromosome elements take on a ‘zig-zag’ pattern, but the SMC3 signal reduces significantly as the SYCP1 signal is lost, in keeping with zygotene where the axial element signal is not observed without a central element signal. We consistently observed two different sized spherical SMC3 positive regions, both of which were DAPI negative (Fig. 6, red and yellow arrows).

### During diplotene platypus SCs rapidly contract and the axial cores and central elements concurrently disassemble

During diplotene the murine central elements begin to dissolve while the axial elements persist and the desynapsed chromosomes are joined only by chiasmata^36^. In contrast to this, we never observed desynapsing SCs in platypus. Instead the axial cores appear to compress forming a zig-zag pattern as the central elements and axial cores dissolve (Fig. 6) and the sex chromosome specific SMC3 loading is lost. Contrasting this, the mouse X and Y chromosomal axial elements maintain SMC3 (Fig. 6) while the autosomes show limited SMC3 foci, as previously reported in mice for SMC3^37^ and for other cohesin components^17^. In platypus SMC3 also showed limited weak foci on all chromosomes, however, it was common to observe SYCP1 staining directly adjacent to some strong SMC3 foci (Fig. 6, blue arrows).

### Differential SMC3 loading on paired and unpaired regions of platypus sex chromosomes at prophase I

To better characterise the sex chromosome specific SMC3 accumulations we immunostained for SMC3 in combination with DNA FISH using a selection of BACs and chromosome painting probes targeting XY specific and XY shared (PAR) regions. After SMC3 immunostaining, imaged cell coordinates were recorded prior to a series of DNA FISH experiments using combinations of X_1_ pooled, X_4_, Y_5_ and pooled PAR probes (Table 1). During early prophase I, the XY specific probes hybridised to chromosomal DNA which had axial element SMC3 but lacked a central element (SYCP1) (Fig. 7). At mid pachytene the XY specific probes colocalised with paranucleolar SMC3 but from diplotene associated with protein bodies or had no association with any SC signal (Fig. 7). By contrast, from zygotene through to diplotene, probes targeting XY shared regions (PARs, green arrowheads) localised to elements with cohesin and fully formed central elements evidenced by SYCP1 positive staining (Fig. 7).

**Table 1:** Probes used in DNA FISH experiments*.

Probe Name	Chromosome	X/Y specific or PAR	Paper first cited
CH236-804 O01	X_1_	Specific	—
CH236-378 F21	X_1_	Specific	Veyrunes, *et al*.^53^
CH236-200 G3	X_1_	Specific	Veyrunes, *et al*.^53^
CH236-341 C3	X_1_	Specific	Veyrunes, *et al*.^53^
CH236-286 H10	X_1_Y_1_	PAR	Veyrunes, *et al*.^53^
CH236-78 K11	X_2_Y_2_	PAR	Veyrunes, *et al*.^53^
Oa_Bb-145 P9	Y_2_	Specific	—
CH236-158 M16	X_3_	Specific	Veyrunes, *et al*.^53^
Oa_Bb-462 C1	X_3_Y_3_	PAR	Tsend-Ayush, *et al*.^56^
Oa_Bb-397 I21	Y_3_	Specific	—
CH236-639 O23	Y_3_X_4_	PAR	Veyrunes, *et al*.^53^
Y4 paint	Y_4_	Specific	Grutzner, *et al*.^27^
Oa_Bb-466 A15	Y_4_X_5_	PAR	—
CH236-820 A16	X_5_	Specific	Veyrunes, *et al*.^53^
CH23-634 B19	X_5_	Specific	—
CH236-830 M18	X_5_	Specific	—
CH236-236 A5	X_5_	Specific	Veyrunes, *et al*.^53^
CH236-752 F12	X_5_	Specific	Veyrunes, *et al*.^53^
Oa_Bb-152 P15	Y_5_	Specific	Tsend-Ayush, *et al*.^56^

^*^Probes were purchased from two libraries, the female platypus library held at the Children’s Hospital Oakland Research Institute (CH236) and the male platypus library from the CUGI BAC/EST Resource Centre, Clemson, South Carolina USA (Oa_Bb).

**Figure 8:**
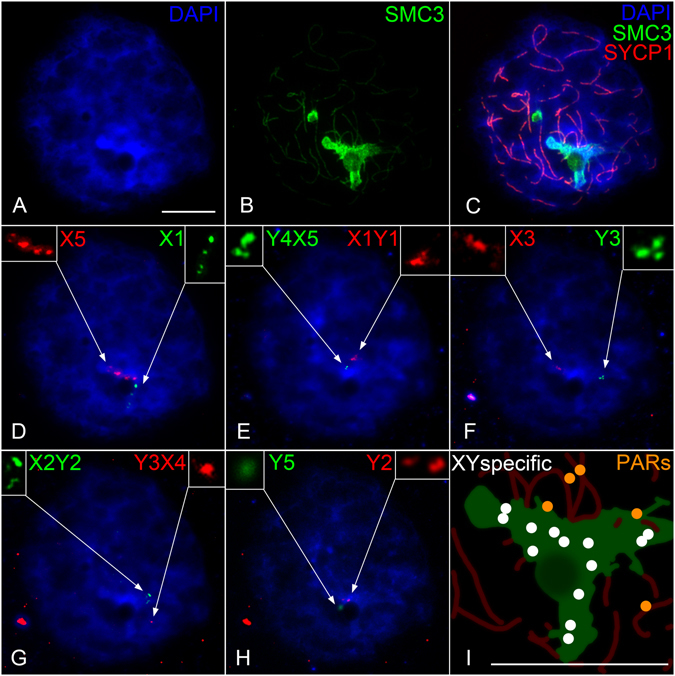
Sequential sex chromosome specific immuno-FISH on a platypus “stage g” pachytene cell. Nuclei were stained with SYCP1 (red), SMC3 (green) and DAPI (blue) (**A**–**C**). Sequential FISH were carried out in five subsequent experiments in the order **D**–**H** where enlarged views of the FISH signal are shown in the top corners of each panel (**D**–**H**). A schematic of an enlarged view of region containing sex chromosomes indicates the location of XY specific probes (white) and PARs (orange) (**I**). Scale bars = 10 µm.

As XY specific sequences localised to regions of maximal SMC3 loading, we aimed to ascertain the localisation of additional XY specific regions in relation to the different SMC3 staining intensities. In a separate immuno-FISH experiment, we carried out serial BAC DNA FISH in five consecutive experiments on the same cells to determine the location of almost the entire chain at mid pachytene relative to SMC3 in the same nucleus. The experiments were carried out by applying BAC probe sets in the following order: a) five X_5_ BACs and four X_1_ BACs, b) X_1_Y_1_ [PAR1] and Y_4_X_5_ [PAR8], c) X_3_ and Y_3_, d) Y_3_X_4_ [PAR6] and X_2_Y_2_ [PAR3], e) Y_2_ and Y_5_. This revealed that the X and Y specific sequences (X_1_, Y_2_, X_3_, Y_3_, X_5_ and Y_5_) consistently colocalised with the intense SMC3 domain which lacked central element staining (Fig. 8(D), (F), (H) and (I)). In contrast, all PAR signals (X_1_Y_1_, X_2_Y_2_, Y_3_X_4_ and Y_4_X_5_) were positioned either outside or on the periphery of the SMC3 domain and in all cases had associated central element staining (Fig. 8(E), (G) and (I)).

## Discussion

This is the first detailed analysis of the organisation at male meiotic prophase I of the most complex known mammalian sex chromosome system. We observed massive and differential recruitment of cohesin specifically to unpaired parts of the sex chromosome chain and this specific and transient cohesin accumulation to the sex chromosome complex coincided with nucleolar association and progressive condensation. There were clear differences between platypus and mouse in the temporal and spatial cohesin accumulation dynamics including the rapid loss of SMC3 from specific regions of sex chromosomes. The relationship between these differences and our recent reports on the highly diverged platypus SYCP3^31^ and the absence of platypus MSCI^32^ currently remain unclear in terms of pairing checkpoints or preparations for metaphase and segregation of the 10 sex chromosomes.

### Platypus sex chromosomes exhibit differential cohesin loading on sex chromosomes

In platypus we observed a remarkably different XY cohesin recruitment programme in terms of the extent and specificity to unpaired regions which deviates significantly from that described for other species. Although there are exceptions, the majority of studied species show cohesin sex chromosome loading at pachytene as weaker, as for REC8 in mouse, or comparable to autosomes, such as STAG3 or SMC3^38^. In some cases the former may simply be due to the fact there is only one chromosomal axis on the sex chromosomes whereas the signal observed between autosomal homologues is created from the close proximity of two axial cores^39–41^. Such a phenomenon was also observed for grasshopper B chromosomes^42^. In addition, generalisations are confounded by differing cohesin subunit loading dynamics occurring on AEs following pachytene^38^. Interestingly, in grasshopper preleptotene cells the X chromosome lacks SMC3, however by leptotene the levels on the X are similar to the autosomes and by pachytene the loading appears weaker than that observed on the synapsed autosomes^39^. In contrast, other mammals such as the armadillo have SMC3 signals on the unpaired sex chromosome axial cores which is indistinguishable in intensity from autosomal SMC3^43^. In horse this is more pronounced, where the X shows greater SMC3 recruitment at axial cores than that seen on the autosomes^44^. Contrasting this are observations in rat where cohesins form scattered foci along the axial core length^41^. Despite the evolution of species specific variations in cohesin loading, platypus sex chromosomes stand out by the sheer magnitude of axial core SMC3 accumulation relative to autosomes.

### Distribution of recruited cohesin to paired and unpaired regions of sex chromosomes: A checkpoint avoidance mechanism

In platypus early pachytene, the cohesin subunits SMC3 and STAG3 colocalise with autosomal SCs similar to other species however cohesin is then massively recruited to the unpaired sex chromosome axial cores. Only in platypus is the increased level of cohesin enrichment restricted to unpaired regions of the sex chromosomes. This raises the question of what cohesin may be doing differently on the unpaired DNA in terms of function and/or interactions and invites speculation on roles in protecting unpaired DNA from checkpoint surveillance machinery which may otherwise halt meiotic progression or simply one of facilitating condensation of the sex chromatin. We recently showed that therian-like sex chromosome transcriptional silencing (MSCI) evolved after the divergence of monotremes due to absence of repressive epigenetic marks such as γH2AX and DNA damage repair pathway components on the sex chromosome chain during late pachytene^32^. The association of the condensed sex chromosome chain with the nucleolus in combination with heavy cohesin loading specifically on the unpaired DNA invites the idea that MSCI may not be required to avoid checkpoint arrest. We are unable to test this hypothesis by inducing autosomal inversions or depleting SMC3 in platypus meiosis therefore rendering these ideas untestable. However, the identification of additional antibodies recognising DDR pathway components and detailed epigenetic profiles on the sex chromosomes in platypus may aid in understanding the observed cohesin dynamics in respect to meiotic progression and possibly segregation. It has been shown that NOR chromosomal positioning dictates pachytene nucleolar localisation during spermatogenesis^45^ and we observe here a conserved central nucleolar location due to the ‘interstitial’ NOR on platypus chromosome 6. The fact that in other species such as mouse the nucleolar-sex chromosome association begins later during pachytene may imply a requirement for platypus sex chromosomes association with nucleoli for chaperoning through the critical period of homologue synapsis^21^. Such a requirement is supported by the observation of Daish and colleagues that Y5 closely and consistently associates with the nucleolus from the earliest pachytene stages^32^ prior to chain contraction (see below) however this association was highly likely but also showed exceptions at early stages of pachytene as seen in Fig. 7. Cohesin has been reported present at centromeric heterochromatin following recruitment by Suv4-20h2^46^ and also at the mating-type heterochromatic region of fission yeast where it is recruited by Swi6(HP1)^47^.

Another area which requires investigation is the mechanism underlying the alternate segregation of the 10 sex chromosomes, an event which may be contingent on the sex chromosome specific accumulation and/or modification of structural complexes incorporating elements of both cohesin and synaptonemal complex components. There are examples to support such speculation in species with achiasmate sex chromosomes which utilise SC structural components to mediate faithful segregation^38, 48, 49^. Nucleolar association is required for establishment of X chromosome inactivation in somatic cells^50^ demonstrating strong functional links between heterochromatin, cohesins, the nucleolus, and sex chromosomes, however the significance of this in the context of platypus meiosis remains unclear and currently is functionally untestable.

### Cohesin may promote sex chromosome self association of the platypus sex chromosomes

Our results show that reorganisation of SMC3 distribution coincides with sex chromosome chain condensation during pachytene. In birds the ZW pair undergoes synaptic adjustment and equalisation^51, 52^ which possibly represents an ancestral mode of organising heteromorphic sex chromosomes that predates the advent of sex chromosome specific silencing (MSCI). Interestingly, while monotremes and birds have sex chromosomes that share significant homology^27, 53^ the heteromorphic chromosomes experience meiosis in males and females respectively. An example where proteins normally function to mediate homologous synapsis having roles in heteromorphic sex chromosome associations is seen in marsupials where SC proteins form a structure called the dense plate. The dense plate appears to mediate and maintain sex chromosome associations in the absence of a PAR^54^ and therefore further highlights the dynamism and functional flexibility of the meiotic machinery following sex chromosome evolution. To our knowledge however this is the first report showing such a marked association of SMC3 on XY specific chromatin.

**Figure 9:**
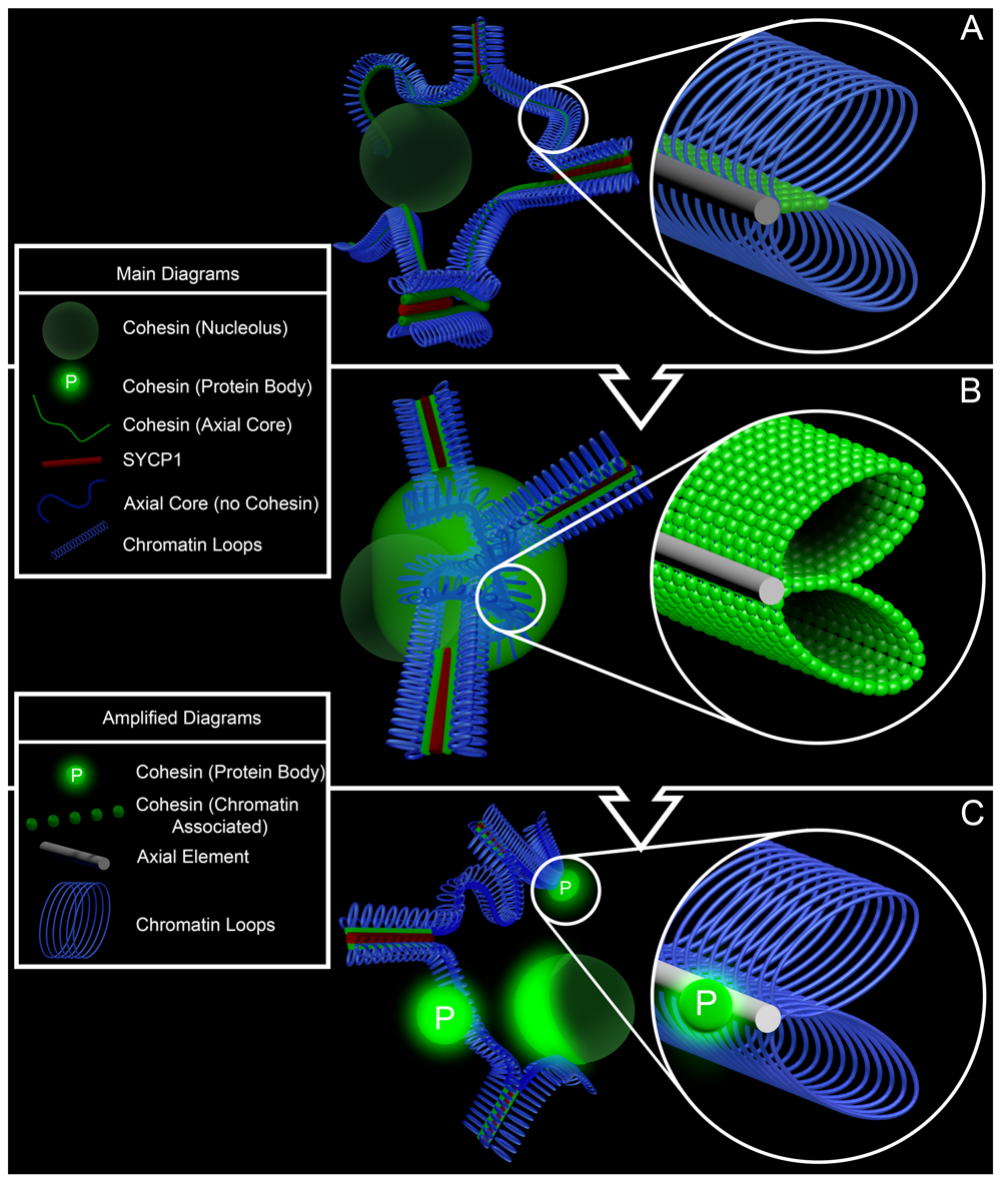
Schematic of platypus sex chromosome and cohesin dynamics during pachytene. In early pachytene (**A**), the sex chromosome chain is attached at one end to the large nucleolus and is spread throughout the nucleus. At this stage greater cohesin loading is observed on the chromosomal axes of the asynaptic regions of the chain. At mid-pachytene, the asynaptic regions of the sex chromosomes are located at a paranucleolar region and at this stage it appears that the SMC3 is recruited to the chromatin loops of the unpaired regions of the chain. By late pachytene, the sex chromosomes begin to spread back out into the nucleus and SMC3 is removed not only from the chromatin loops, but also the axial cores and relocates into protein bodies. The protein bodies spread out in the same direction as the sex chromosomes indicating the protein bodies are physically connected to the sex chromosomes, thus we have depicted an attachment with the chromosomes axial core. Note that in all stages, the sex chromosome chain is attached to the nucleolus which maintains a relatively central location in the nucleus. Also, at all times the PARs form a normal synaptonemal complex and do not accumulate SMC3 onto their chromatin loops at any stage.

In summary, we have shown that organisation of the platypus sex chromosome complex during prophase shows distinct and dynamic cohesin loading that is differentially loaded between paired and unpaired parts of the sex chromosome chain, for which we offer a simplified and functionally speculative model (Fig. 9). Key points of significance include (1) the sex chromosome chain being tethered at one end to a large nucleolar structure with massive cohesin loading on the asynaptic axes relative to autosomes (Fig. 9), (2) the contraction of this cohesin laden asynaptic sex chromatin mass to the paranucleolar region at mid pachytene, and (3) the sex chromosome chain decondenses and spreads throughout the nucleus maintaining nucleolar attachment prior to the cohesin being rapidly removed from both the chromatin loops and axial elements of the XY specific regions at late pachytene (Fig. 9B). The differential cohesin distribution appears highly specific and regulated suggesting an important role in the organisation and positional dynamics of the unique platypus sex chromosome complex.

## Methods

### Specimen Collection

The authors confirm that all experiments were performed in accordance with relevant guidelines and regulations at the University of Adelaide. All experimental approaches were done according to the University of Adelaide biosafety and ethics committee regulations (Institutional Biosafety Committee, Dealing ID 12713). Platypus samples were collected in 2002 (AEEC permit R.CG.07.03, Environment ACT permit LI 2002 270, NPWS permit A193) and 2008 (AEC permit no. S-49-2006) at the Upper Barnard River (New South Wales, Australia) during the breeding season. Mouse testis were obtained from three week old animals (Swiss white).

### Storage Preparation

The method of meiotic sample preparation and storage was the same as previously described by Daish *et al*.^30^. Briefly the testis were placed in a petri dish containing 1 x PBS with 1 x Protease Inhibitor (Roche). The surrounding tunica albuginea was punctured and the testis material was teased apart using needles resulting in the release of meiotic cells and sperm from the ruptured tubules. A pipette was used to break up and flush the remaining material to ensure maximal release of cells. The resulting cell suspension was mixed with DMSO (to a final concentration of 10%), slow cooled and stored in liquid nitrogen for future use.

### Meiotic Spreads

Meiotic spreads of testis material were prepared by a method based on that previously described by Peters *et al*.^55^. Briefly, a 75 µL aliquot of the testis 10% DMSO suspension was thawed. Either 75 µL 0.3 M sucrose or 75 µL of 75 µM KCl was added to the testis material and left to sit at room temperature for 5 minutes. Next 50 µL of 1% PFA/0.15% Triton X-100 pH 9.5 was dropped onto slides and spread with a pipette tip. 20 µL of the testis material/hypotonic solution was then dropped onto the PFA coated slides. Slides were kept in a moist chamber for 2 hours and then washed in 50 mL of 1 X PBS followed by a final wash with water and wetting agent. Slides were air dried and used immediately for immunostaining or stored at −20 °C.

### Immunostaining

Immunostaining was carried out as per Schoenmakers *et al*.^52^. Briefly, slides were blocked in 1 X PBS with 0.5% w/v BSA and 0.5% w/v milk powder three times for five minutes and incubated in a humid chamber with a primary antibody (1:200 dilution) in 1 X PBS/10% w/v BSA for either 2 hours at 37 °C or overnight at room temperature. Slides were then washed in 1 X PBS three times for five minutes each and blocked in 10% v/v goat serum in blocking buffer for three times for five minutes each and incubated at 37 °C for 2 hours in a humid chamber with a secondary antibody (1:400 dilution) in 10% v/v goat serum in blocking buffer (5% w/v milk powder in 1 X PBS centrifuged at 13,200 for 10 minutes). Finally slides were washed three times for five minutes in 1 X PBS, dipped in DAPI (1:5000 dilution) for one minute, washed twice with MQ water and mounted with Vectashield (Vecta Laboratories).

### Antibodies

To observe the central element we used an antibody (Novus, NB300-229) raised against the central element protein; Synaptonemal Complex Protein 1 (SYCP1). We employed the use of an SMC3 antibody (Abcam, Ab9263) to visualise the axial core in platypus since it has been previously shown that SMC3 recruitment follows synaptic progression^39^.

### DNA FISH

A method of Fluorescence *In Situ* Hybridisation (FISH) based on the original protocol described by McDougall *et al*. (1972) was optimised for PFA fixed meiotic material. Briefly, BAC DNA probes were directly labelled using Klenow and a random 9mer primer overnight with either SpectumOrange or SpectrumGreen 2′deoxyuridine-5′-trisphosphate (Abbott Molecular). To reduce the background signal on slides, labelled probes were subsequently co-precipitated with salmon sperm and sonicated platypus male genomic DNA. Pellets were dissolved in deionised formamide and 2 X hybridisation buffer containing dextran sulfate. Slides containing the PFA fixed meiotic material were dehydrated in an alcohol series (70–100% ethanol) for 5 minutes each, treated with RNase A (100 µg/mL) for 30 minutes at 37 °C, pepsin (to a final concentration of 0.005%) for 10 minutes at 37 °C, fixed in formaldehyde (final concentration of 1%) in PBS/50 mM MgCl_2_, dehydrated in an alcohol series (70–100% ethanol) for 5 minutes each, denatured in 70% formamide/2 X SSC at 70 °C for 7 minutes for the first FISH and 3 minutes for each subsequent FISH on the same slide, dehydrated (70–100% ethanol) for 5 minutes each and air dried ready for hybridisation with the labelled probe. The probes were applied to the dry slide and covered with a coverslip, sealed with rubber cement and incubated overnight at 37 °C in a moist chamber. The coverslip was removed and slides were washed three times in 50% formamide/2 x SSC followed by 2 x SSC, once in 2 X SSC at 42 ^o^C for 5 minutes, once at 60 ^o^C in 0.1 X SSC for 5 minutes and finally once at 42 ^o^C in 2 X SSC for 5 minutes. Slides were then stained with DAPI for one minute, washed twice with MQ water and mounted with Vectashield (Vecta Laboratories).

### DNA FISH Probes

#### Image Acquisition and Processing

Slides were visualised using a Zeiss AxioImager 2.1 microscope equipped with a 10x ocular and 10x, 20x, 63x and 100x objective lenses. Fluorescent tags were visualised using 3 filters: DAPI, for DAPI stained DNA; GFP, for SpectrumGreen and Alexa 488 and; DS red, for SpectrumOrange and CY3. Images were recorded via an Axiocam CCD-camera and Zeiss Axiovision software. Unless otherwise stated, images were captured using the 100x objective lens, appearing as 1000x with the 10x ocular. Images were processed using Axiovision (Zeiss), GIMP 2.8 (http://www.gimp.org/) and ImageJ (National Institutes of Health, United States; http://rsb.info.nih.gov/ij).

## Electronic supplementary material

Supplementary Data
