# Supplementary material for: Differential cohesin loading marks paired and unpaired regions of platypus sex chromosomes at prophase I
Source: Sci Rep. 2017 Jun 26;7:4217. doi: 10.1038/s41598-017-04560-5 (PMC5484699; doi:10.1038/s41598-017-04560-5)
Supplement: Supplementary file 1 — Supplementary Data [file 41598_2017_4560_MOESM1_ESM.doc]

**Title**

Differential cohesin loading marks paired and unpaired regions of platypus sex chromosomes at prophase I

Aaron E. Casey^1^, Tasman J. Daish^1^, Jose Luis Barbero^2^ and Frank Grützner^1*^

^1^The Robinson Research Institute, School of Biological Sciences, the University of Adelaide, South Australia.

^2^ Centro de Investigaciones Biologicas (CSIC)/ Ramiro de Maeztu, 9 28040 Madrid, Spain

*correspondence, email: frank.grutzner@adelaide.edu.au

Supplementary Figures:


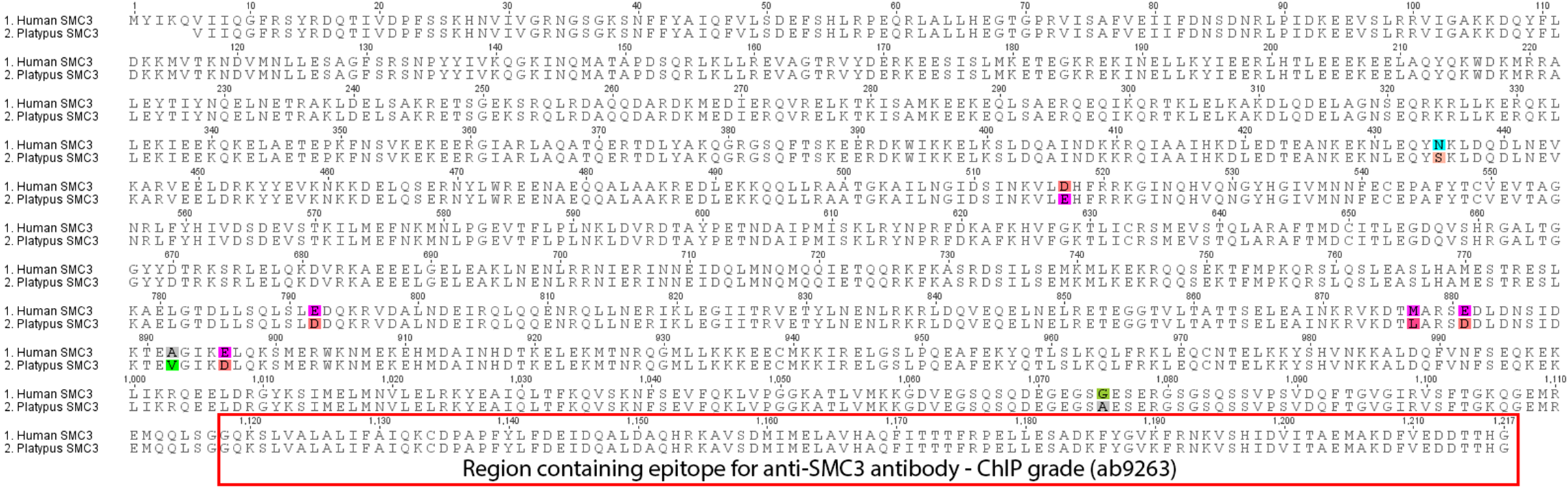


Figure S1. Alignment of human and platypus SMC3 protein sequences. Sequences were aligned in Geneious using MAFFT with standard alignment options. Sites that are not identical are highlighted. The first exon of platypus sequence has yet to be identified and is missing from the alignment. The region containing the human epitope used to generate the antibody used in this study is marked with a red box, where there is 100% conservation between the two sequences.


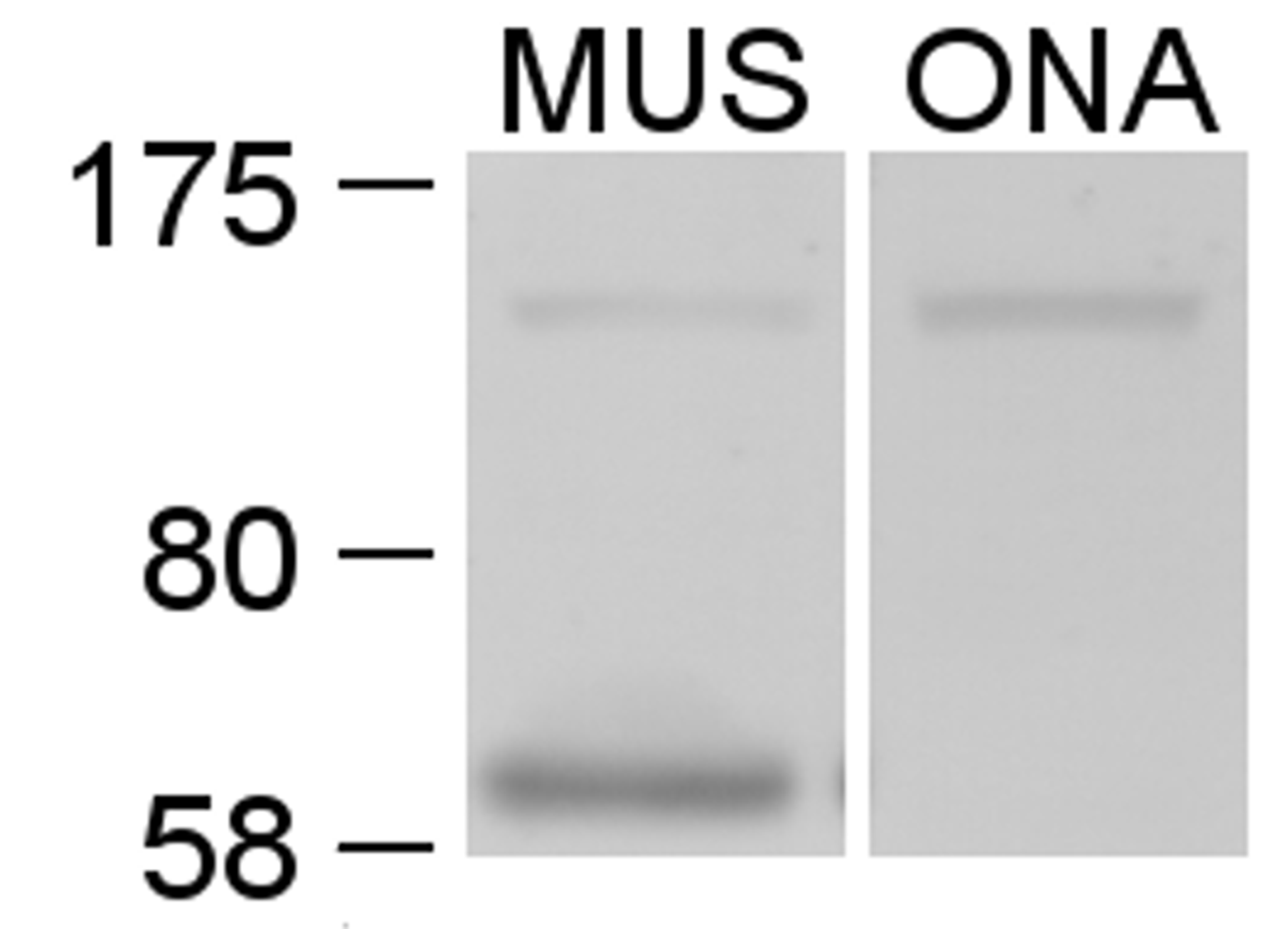


Figure S2. Western blot of platypus and mouse testis SMC3. Nuclear extracts from mouse (MUS) and platypus (ONA) testis were subjected to immunoblot analysis using the polyclonal SMC3 antibody. In platypus and the mouse control, a band is observed at the expected size of 140kDa. The mouse control also shows an unidentified second band at ~60kDa.


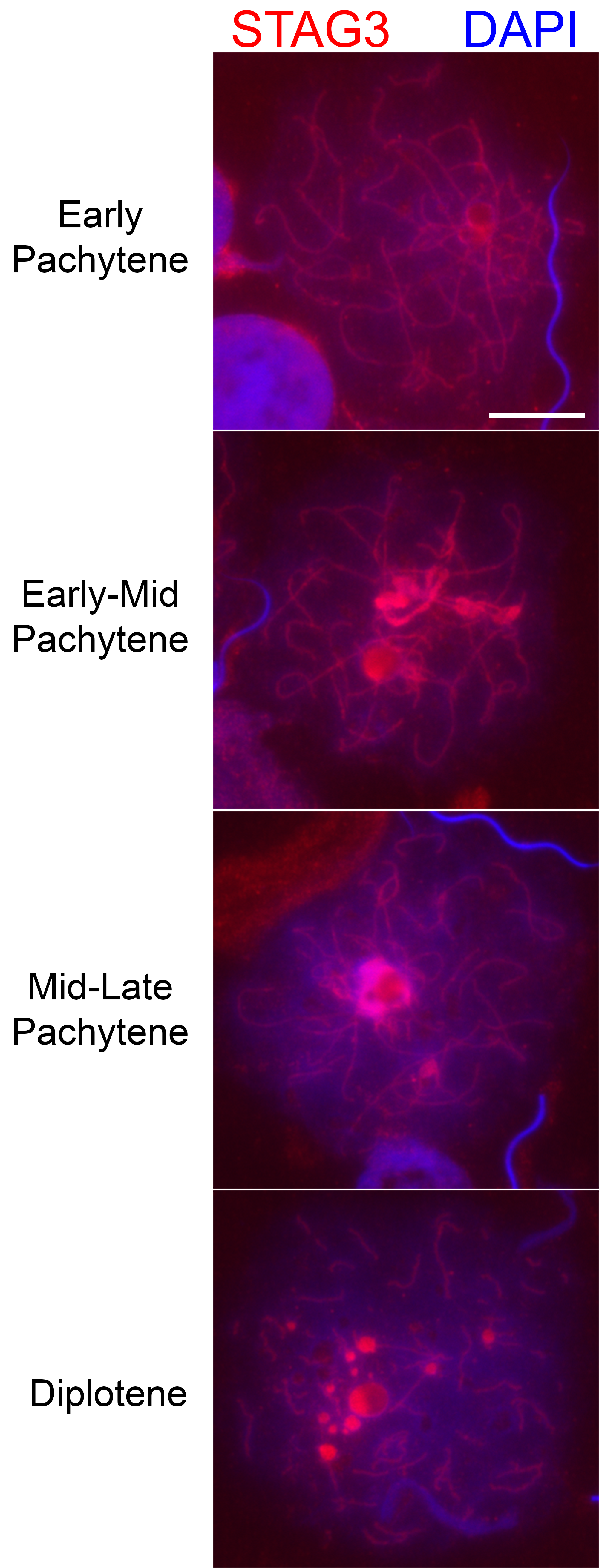


Figure S3. Immunostaining patterns of STAG3 in platypus prophase I cells. Stages from early pachytene through to diplotene are represented. The patterns observed match those observed for SMC3 immunostained platypus prophase I cells.
